# Testing a discrete choice experiment including duration to value health states for large descriptive systems: Addressing design and sampling issues

**DOI:** 10.1016/j.socscimed.2014.05.026

**Published:** 2014-08

**Authors:** Nick Bansback, Arne Risa Hole, Brendan Mulhern, Aki Tsuchiya

**Affiliations:** a School of Population and Public Health, University of British Columbia, 2206 East Mall, Vancouver, BC V6T 1Z3, Canada; b Centre for Health Evaluation and Outcome Sciences, St Paul's Hospital, Vancouver V6Z 1Y6, Canada; c Department of Economics, University of Sheffield, Sheffield S1 4DT, UK; d Health Economics and Decision Science, School of Health and Related Research, University of Sheffield, Regent Court, Sheffield S1 4DA, UK

**Keywords:** UK, Health-state valuation, Discrete choice experiment, EQ-5D-5L

## Abstract

There is interest in the use of discrete choice experiments that include a duration attribute (DCE_TTO_) to generate health utility values, but questions remain on its feasibility in large health state descriptive systems. This study examines the stability of DCE_TTO_ to estimate health utility values from the five-level EQ-5D, an instrument with depicts 3125 different health states. Between January and March 2011, we administered 120 DCE_TTO_ tasks based on the five-level EQ-5D to a total of 1799 respondents in the UK (each completed 15 DCE_TTO_ tasks on-line). We compared models across different sample sizes and different total numbers of observations. We found the DCE_TTO_ coefficients were generally consistent, with high agreement between individual ordinal preferences and aggregate cardinal values. Keeping the DCE design and the total number of observations fixed, subsamples consisting of 10 tasks per respondent with an intermediate sized sample, and 15 tasks with a smaller sample provide similar results in comparison to the whole sample model. In conclusion, we find that the DCE_TTO_ is a feasible method for developing values for larger descriptive systems such as EQ-5D-5L, and find evidence supporting important design features for future valuation studies that use the DCE_TTO_.

## Highlights

• DCE with duration attribute (DCE_TTO_) used to value EQ-5D-5L with 3125 health states.
• DCE_TTO_ generated consistent health state values for this large descriptive system.
• Some evidence of small fatigue effects when administering more than 10 DCE_TTO_ tasks.
• Offers guidance on balancing number of tasks per respondent and total sample size.

## 1 Introduction

In the absence of suitable market data, stated preference surveys have become an important source of data for informing health policy. In these surveys, choices over hypothetical scenarios are used to elicit individuals' preferences for the attributes making up those scenarios. Two crucial design aspects to these surveys are: the type of preference elicitation technique, and the number of choices each respondent faces.

For policy decisions regarding the cost-effectiveness of health care interventions, there is much debate over the most suitable type of health state valuation technique to use. Conventional approaches have focussed on choice based techniques such as the Time Trade Off (TTO) and the Standard Gamble, whereby a value for each health state is identified for each respondent through an ‘iterative’ procedure that homes in on a point of indifference. However, both techniques have several shortcomings. First, they can be cognitively challenging (Patrick et al., 1994; Dolan et al., 1996) generating individual responses that are either logically inconsistent or otherwise difficult to accept at face value (e.g. all health states have the same value) (Craig and Ramachandran, 2006). These observations are typically excluded from analysis, which would potentially reduce the representativeness of the sample. Second, the iterative administration introduces biases because of the pathway through which values are elicited (Ternent and Tsuchiya, 2013), leading to distributions of values that are discontinuous (with a gap between the best and next best state; Stalmeier et al., 2005) and bimodal (for mild/moderate states and severe states). Third, the treatment of negative values can be arbitrary and controversial (Lamers, 2007). The exception to this last point is the introduction of “lead time” in TTO (Devlin et al., 2011), but this method has its own problems, such as a framing effect caused by the lead time (Devlin et al., 2013).

As a consequence, there has been a growing interest in developing alternative valuation techniques. One focus of research has been on the use of ordinal preferences using ranking (McCabe et al., 2006), Discrete Choice Experiments (DCEs) (Ryan et al., 2006; Rowen et al., 2012) or Best Worst Scaling (Coast et al., 2008). Since the DCEs are based on random utility theory, it models peoples' observed choices assuming they include error. In other words, it is more robust and can accommodate respondents making mistakes in the valuation exercise. Further, some biases introduced by the particular ordering in which preferences are elicited by an iterative procedure can be avoided. Finally, while DCE values are estimated on an unobserved and arbitrarily anchored latent scale, they can be anchored on the health utility scale with 1 for full health and 0 for dead by incorporating duration as an attribute of the DCE (Bansback et al., 2012; Flynn, 2010; Norman et al., 2013; Viney et al., 2014). This task closely resembles the conventional TTO (and is thus referred to as the DCE_TTO_), but does not require a separate task or data manipulation for states considered worse than dead.

While the DCE_TTO_ appears to be a promising technique for use in future stated preference surveys, two practical knowledge gaps exist, that this paper aims to fill. First, it is unknown whether the DCE_TTO_ will be feasible in valuing larger descriptive systems. The original DCE_TTO_ (Bansback et al., 2012) valued the three-level version of EQ-5D (Brooks, 1996), which has only 243 possible health states. A majority of health state descriptive systems have more attributes and more attribute levels resulting in considerably larger numbers of plausible health states. Since the DCE_TTO_ compares different plausible health states with different durations, larger descriptive systems lead to exponentially larger experimental designs which may result in impractically large numbers of required valuations. A second related knowledge gap is how many DCE_TTO_ tasks each respondent should be asked. In contrast to the large literature on the statistical design of DCEs (see e.g. Carlsson and Martinsson, 2003) there is more mixed evidence on the optimal number of tasks to allocate to respondents in a choice experiment (Louviere et al., 2013). Often, well developed fractional factorial designs for tasks with reasonable numbers of attributes and levels generate a greater number of choice sets than what is considered feasible to complete for a single respondent, which means that in practice the choice tasks tend to be “blocked” into subsets of the full design, and each allocated to a subset of respondents. If there is a learning effect so that respondents need to try out a few DCE tasks before they can generate stable data, then the blocks will need to be of a certain size. On the other hand, if there is a fatigue effect, then there will be a limit to the size of each block. The decision for the number of tasks to give each respondent therefore is related to the number of respondents.

This paper seeks to establish whether the DCE_TTO_ is an judicious alternative protocol for deriving population value sets for large descriptive systems such as the EQ-5D-5L (Herdman et al., 2011). We do this by examining both empirical observations and practical design issues to investigate further the feasibility of the approach, and inform the design of future DCE_TTO_ studies. The paper begins by describing the survey and design of the DCE_TTO_. The following section describes the methods used to address our two aims. Our first aim is to examine whether DCE_TTO_ can appropriately be used to value a larger descriptive system such as the EQ-5D-5L. We examine four specific objectives including whether the results are consistent, how they compare to previous DCE_TTO_ models, whether respondents trade time and whether there is agreement between ordinal and aggregate values. Our second aim is to explore how best to design the survey considering the number of tasks and respondents. We examine three specific objectives including whether there are learning and/or fatigue effects, and whether using different numbers of tasks per respondent and sample sizes (holding the design and overall number of observations constant) have an effect. The results section is followed by a discussion that summarizes the evidence for the DCE_TTO_ in future studies, and addresses notable limitations.

## 2 Methods

### 2.1 Elicitation task design

**Fig. 1:**
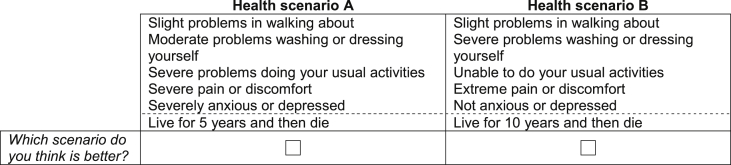
DCE_TTO_ question example.

The DCE_TTO_ task used in this study considers pairs of scenarios, each based on the health states described by the five attributes of the EQ-5D-5L (mobility, self care, usual activities, pain/discomfort and anxiety/depression) and an additional duration attribute. In contrast to the three-level EQ-5D, the five-level EQ-5D-5L adds two intermediate levels of severity (none, slight, moderate, severe, extreme/unable). Each scenario asked a respondent to consider themselves living in a particular health state for one of three levels of duration *T* (where *T* = 1, 5, or 10 years) followed by death. The longest duration was set to 10 years to be commensurate with the standard time frame of the TTO protocol used in the EQ-5D valuation study (Dolan, 1997). The DCE_TTO_ task requires each respondent to select the scenario they prefer. Fig. 1 displays an example of a task used in the study.

### 2.2 Health state scenario selection and allocation

Combining each EQ-5D-5L state with three levels of duration amounts to 9375 possible scenarios and therefore 87.9 m possible DCE_TTO_ scenario pairs (tasks) to choose from. As is explained below, DCE_TTO_ models the pairwise choice data in terms of interactions between the health state and duration. The number of choice tasks for DCEs with two alternatives needs to be at least as large as the number of parameters to be estimated (Orme, 2010). In this case, the maximum number of parameters for DCE_TTO_ of EQ-5D-5L with three categorical levels for duration would be 62 (the sum of: EQ-5D-5L main effects 5 × (5 − 1) = 20; categorical duration main effects 3 − 1 = 2; and interactions 20 × 2 = 40). To further increase confidence in the parameter estimates, we selected 120 tasks based on a D-efficient design derived using the modified Fedorov algorithm (e.g. Carlsson and Martinsson, 2003; Kuhfeld, 2005). The D-efficiency was calculated assuming that the true model is a conditional logit model with zero priors for the coefficients. Ten different designs were created, based on different random starting points for the design algorithm, and found to have comparable efficiency levels. One design included a higher number of tasks (18 (15%)) where duration differed between the scenarios, and therefore was selected for use in the study. The EQ-5D-5L may produce implausible health state combinations (for example extreme problems with mobility but no problems with usual activities), and it would be possible to use a restricted design to avoid these. However, we used an unrestricted design, and checked the states selected for implausible combinations of attribute levels, but found none.

### 2.3 Survey design

The DCE_TTO_ study was administered using an on-line survey. Each respondent completed 15 DCE_TTO_ tasks across three experimental “modules”, each made up of five tasks. The survey had 36 “versions”, so a total of 108 modules to fill. Firstly, the 120 tasks selected by the D-efficient design were split into 24 “blocks” of five tasks, where each scenario pair appeared once. Following this another 12 “blocks” were generated, where 60 of the tasks, including the 18 where duration differed across the scenarios, were randomly allocated. These 36 blocks were used and to fill the 108 modules across the 36 versions, a given block appeared in three different versions, each in a different position.

The 36 blocks were allocated across the 36 versions so that, where appropriate, the data could be analysed in three different “batches”. This enabled us to compare the results across different sample size and task number combinations, keeping the design and the number of observations constant. Table 1 gives a stylised representation of the design. Panel (a) represents the entire data. Each row represents six of the 36 versions, or in other words a sixth of the whole sample, and each column corresponds to one DCE task, grouped into three modules of five tasks. Assuming a sample size of 1800, each cell corresponds to 300 respondents answering one DCE_TTO_ task, whereas the whole grid represents 27,000 DCE_TTO_ observations.

The first set of batches is “single-module batches” and consists of five DCE_TTO_ tasks per respondent, based on one Module across all respondents. Therefore, each single-module batch would total 9000 DCE_TTO_ observations. These are called “Module1 (All)”, “Module2 (All)” and “Module3 (All)”, depending on which Module the data come from. These single-module batches are illustrated in Table 1 (b).

The second set of batches is “double-module batches” and comprises 10 DCE_TTO_ tasks per respondent, based on Modules 1 and 2 of half the respondents. Each double-module batch has 900 respondents, and includes 9000 observations. Two such batches are possible depending on which half of the sample, and these are called “Module12 (B1)” and “Module12 (B2)”. These double-module batches are illustrated in Table 1(c).

The third set of batches is “triple-module batches” and contains 15 DCE_TTO_ tasks per respondent, based on Modules 1, 2, and 3 of a third of the respondents. Each triple-module batch has 600 respondents providing 9000 observations. Three such batches are possible, referred to as “Module123 (B1)”, “Module123 (B2)” and “Module123 (B3)”. These triple-module batches are illustrated in Table 1(d). Note that the total number of DCE_TTO_ observations (9000) and the make-up of the tasks (180) are kept constant across all the above batches.

Finally, the whole dataset “ALL” (*n* = 1,800, 15 DCE_TTO_ tasks each, 27,000 observations) is in Table 1(e).

### 2.4 Recruitment and the sample

Respondents were recruited from an existing commercial internet panel, and were selected according to quotas based on the UK general population for age (across five age groupings 18–24; 25–34; 35–44; 45–54; 55–65) and gender. Invitations were sent out by e-mail, and if the respondent and respondents clicked a link to access the survey. They then read detailed project information and consented to take part. If the quota category a potential respondent belonged to was full, they were screened out prior to entering the survey. Respondents were also screened out if they completed the survey in less than the minimum completion time of 3 min.

Respondents entering the survey firstly completed demographic and self reported health status, health and life satisfaction questions, and EQ-5D-5L for their own health. They were then presented with information about the DCE_TTO_ tasks including the attributes used, and instructed to imagine that they would experience each health state for the period shown without relief or treatment, that death would be very swift and completely painless, and that they would have no other health problems besides what was indicated. A practice task was then completed, followed by the three question modules. The survey was designed and hosted by the market research company. Ethical approval was obtained from the University of Sheffield, School of Health and Related Research Ethics committee.

### 2.5 Analysis

To determine the coefficients for the DCE_TTO_ the analysis described in Bansback et al. (2012) is followed. Briefly, the utility *μ* of each respondent *i* is defined to be a function between a vector of levels for each EQ-5D attribute ***x*** and life years *t* in each scenario *j* so that:

(1) $$\mu_{ij}=\alpha +\beta t_{ij}+\lambda^{\prime }x_{ij}t_{ij}+\in_{ij}$$

Of these, the constant *a* can be included to examine left/right bias, but is expected to be equal to zero; *β* represents the value of living in full health for the specified duration and is expected to be positive; *λ* represents the disutility of living with the specified set of EQ-5D-5L health problems for the same duration and thus is expected to be negative; and *ε*_*ij*_ is a random term which is assumed to be IID extreme value type 1 distributed. Duration is treated as continuous and conditional logit regression is used to estimate the coefficients.

Bansback et al. (2012) show that the values for each individual health state can be anchored on the health utility scale (V) by solving a conventional TTO paired comparison where living in full health for *t* years is equivalent to living in health state *x* for 10 years using the estimated coefficients from (1). This is analogous to Norman et al. (2013) and Viney et al. (2014) and can be solved as:

(2) $$V_{j}=1+\frac{{\hat{\lambda }}^{\prime }}{\hat{\beta }}x_{j}$$

The value of a health state is expressed in two arguments: the value of full health and the disutility determined by EQ-5D-5L. For the state of full health, ${\hat{\lambda }}^{\prime }$***x***_*j*_ = 0 and so *V* = 1. A health state equivalent to being dead can be conceived as one where the disutility associated with the state (${\hat{\lambda }}^{\prime }$***x***_*j*_) exactly cancels out the utility associated with full health ($\hat{\beta }$), so that ${\hat{\lambda }}^{\prime }x_{j}/\hat{\beta }$ = −1 and therefore *V* = 0. If the state is severe, then the absolute value of ${\hat{\lambda }}^{\prime }$***x***_*j*_ may exceed $\hat{\beta }$, or in other words, the magnitude of the disutility associated with the state may be larger than the difference between full health and being dead. If so, this would result in a negative *V*, implying a state worse than being dead.

Note that the anchoring of the utility function for dead at 0 is achieved through the relative size of the two regression coefficients *β* and *λ* in equation (1) above, and does not require the inclusion of the state of being dead in the DCE_TTO_, or as a supplementary question. The anchoring of the utility function for full health at the value 1 is achieved through equation (2): since ${\hat{\lambda }}^{\prime }$***x***_*j*_ = 0 for full health, equation (2) anchors full health at whatever value given in the first argument.

Objective 1 To determine if the DCE_TTO_ can produce logically consistent values for EQ-5D-5L, with more detailed levels than the three-level EQ-5D.

Coefficients for each attribute were compared to identify if worse levels had lower values. Two visual inspections were used. First, the anchored coefficients grouped by attribute were plotted on a graph alongside their confidence intervals. Second, a histogram of the 3125 predicted values was drawn.

Objective 2 To compare the consistency of the level coefficients for each attribute with those of the original three-level EQ-5D study (Bansback et al., 2012).

The levels of anchored coefficients were compared with a corresponding plot based on the coefficients from the original three-level EQ-5D study.

Objective 3 To assess the impact of adding the duration attribute to a DCE, by examining how respondents traded length of time for quality of life, i.e. whether respondents chose the scenario with a shorter duration.

Three additional analyses were performed to assess whether respondents trade time when presented with scenarios where duration differs. Firstly, time trading behaviour was explored by examining the frequencies of respondents who were willing to trade time when presented with a task where duration differed between the scenarios. The proportions of respondents choosing the shorter duration was investigated irrespective of the EQ-5D-5L state presented. Second, the proportion of respondents who sometimes chose the longer and sometimes chose the shorter duration was examined, where the pairs presented allowed us to investigate this. Third, trading behaviour was assessed in relation to the utility value associated with each health scenario included in the task where duration differs.

Objective 4 To explore the extent of agreement between individual ordinal preferences and aggregate cardinal values.

The difference in the value of the health scenario in terms of Quality Adjusted Life Years (QALYs) was calculated across the 120 tasks, to represent the aggregate cardinal values. The value for each scenario is based on the predicted value of the EQ-5D-5L state multiplied by the specified duration. The differences in QALYs across tasks were then compared to the proportion of respondents choosing each scenario. If the majority chose the health state scenario with the lower predicted QALYs, then this would indicate a “disagreement” between individual ordinal preferences and aggregate cardinal values.

Objective 5 To explore the existence of learning or fatigue effects, i.e. whether respondents answer the choices at the beginning of the experiment less or more consistently than the choices towards the end.

First, the predicted values obtained from models estimated on the three single-module batches Module1 (All), Module2 (All) and Module3 (All) are compared against each other, and against the predicted values for the whole sample. Since these single-module batches represent the first, second and final modules respondents answered, a divergence in the predictions can be interpreted as evidence of learning and fatigue effects. Second, along the lines of Bradley and Daly (1994), we estimate a model on the full sample in which the scale of the error term is allowed to vary by batch^1^. Since the scale is inversely proportional to the error variance, a decrease in scale towards the end of the choice sequence can be interpreted as a fatigue effect, and vice versa. Furthermore, the likelihood-ratio statistic

$$LR=-2\left(LL_{R}-LL_{U}\right)$$

can be used to test the null hypothesis that the respondents' preferences are stable throughout the choice sequence (Swait and Louviere, 1993). Here *LL*_*R*_ is the log-likelihood of the model estimated on the full sample which allows for scale differences but assumes that *α*, *β* and *λ* do not vary by batch. This restricted model is estimated using the Stata module clogithet (see Hole, 2006a, 2006b). *LL*_*U*_ is the sum of the log likelihoods of the three models estimated on the batch-specific subsamples. Together, these form the unrestricted model, which allows for variations in both scale and preferences by batch. Under the null the test statistic is *χ*^2^ distributed with 40 degrees of freedom (the number of degrees of freedom is given by the number of parameters in the unrestricted model minus the number of parameters in the restricted model).

Objective 6 To compare obtaining more DCE_TTO_ answers from a smaller sample and fewer DCE_TTO_ answers from a larger sample, holding the total number of DCE_TTO_ answers and the design constant.

A visual inspection of values from the single-module batch Module1 (All), the two double-module batches, and the three triple-module batches was performed using scatter plots. The two further single-module batches using later modules cannot be used for this analysis, since in a real survey respondents cannot answer a later module without having answered the earlier modules.

Objective 7 To examine the effect of sample size and the number of observations, holding the design constant.

We further inspected the scatter plots (objective 6) holding the design constant, using batches Module1 (All), Module12 (All), and ALL.

## 3 Results

### 3.1 Sample

Between January and March 2011, 5552 respondents were invited to take part, and 4513 (81%) respondents accessed the survey. Of these, 1183 (26% of those accessing the survey) were turned away because their quota was full, leaving 3330 (74%) to enter the survey. Of these, 1020 (31%) dropped out before reaching the DCE_TTO_ questions (321 at the informed consent stage; 296 at the demographics stage; and 403 at the health and satisfaction questions stage). Of the remaining 2310 who entered the DCE_TTO_ questions, 23, 50, and 33 dropped out during the first, second, and third modules respectively. A further nine completed all the DCE_TTO_ questions but failed to formally sign out from the survey and to be counted. Finally, 396 respondents (17% of those who started the DCE_TTO_ questions) were excluded because they completed the survey in less than the minimum time limit of 3 min. This results in the full data set obtained from 1799 respondents (40% of those accessing the survey), each completing the whole survey in more than 3 min. This amounts to 40% of those accessed the survey; 54% of those who entered; and 78% of those who started the DCE_TTO_ questions.

The 1799 respondents were generally representative of the UK population with 54% female and a mean age of 40 (Table 2). The background characteristics do not differ across batches or versions (not shown). The number of respondents completing each of the 36 survey versions ranged from 43 to 52. The number of observations for each of the 120 tasks ranged from 145 to 309 (as a number of tasks were repeated in more than one block).

Objective 1 DCE_TTO_ coefficients overall

**Fig. 2:**
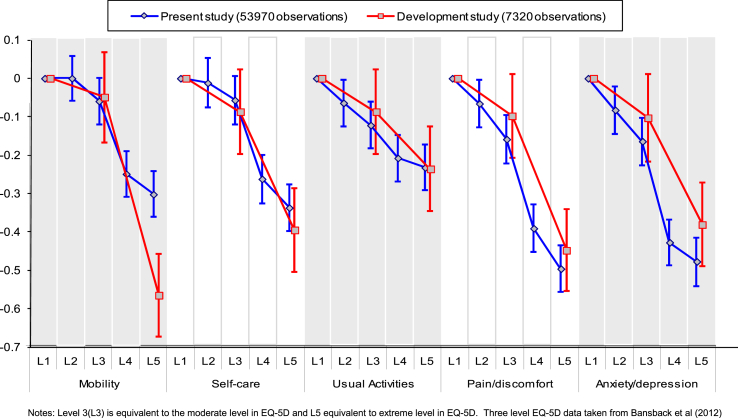
Comparing the anchored coefficients.

**Fig. 3:**
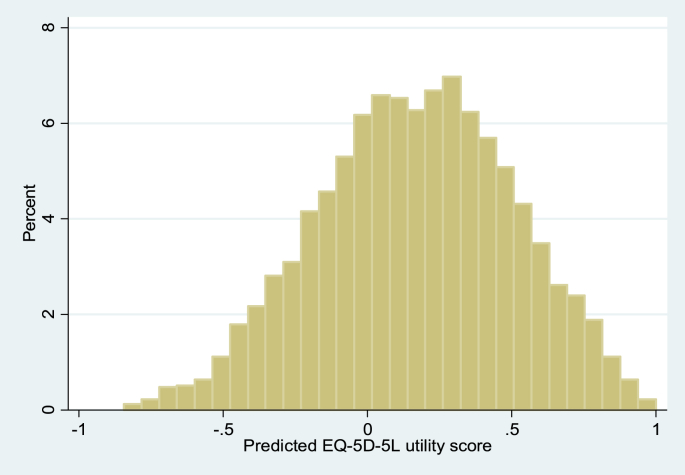
Histogram of all 3125 EQ-5D-5L health state values.

Table 3 reports the unanchored DCE regression coefficients which are based on a model with no intercept. The model with an intercept results in a small but significantly positive intercept. The other coefficients change slightly, but they only have a negligible effect on the anchored coefficients. The positive intercept suggests that there is a bias towards selecting the scenario presented on the left hand side. The coefficient for mobility level 2 interacted with duration (M2xY) does not have the expected sign, but is not significant. All other coefficients are ordered as expected. Fig. 2 depicts the anchored coefficients and confidence intervals. The vertical axis shows the disutility associated with each level within each attribute. The results from the present study are shown by the blue curves. It shows that for example, Mobility level 2 is not statistically significantly different from level 1, and has a positive value indicating that utility increases as health level decreases. Elsewhere, all the curves are downward sloping, indicating that the level coefficients are logically ordered. It also shows that amongst the level 5 coefficients, that for pain/discomfort has the worst disutility, closely followed by anxiety/depression. These two attributes demonstrate a wider gap between levels 3 and 4 than the other three attributes. Fig. 3 displays the distribution of the predicted utility scores for all 3125 EQ-5D-5L health states produced from the anchored coefficients. The value predicted for the worst EQ-5D-5L state (55555) is −0.845, and 31.5% of the 3125 EQ-5D-5L health states have a negative value (i.e. are worse than dead).

Objective 2 Comparison of coefficients with the three-level EQ-5D study

The red curves in Fig. 2 are based on the coefficients for three-level EQ-5D obtained in Bansback et al. (2012), and so along the horizontal axis, the middle level is replaced with level 3 of EQ-5D-5L and the worst level is placed with level 5 of EQ-5D-5L. The major difference between the two sets of coefficients is in the worst level of the Mobility attribute. The middle and worst levels for the Anxiety/depression attribute also fall outside the corresponding confidence intervals. Elsewhere, the level 3 and level 5 coefficients from the five-level model are similar to the level 2 and level 3 coefficients from the three-level model.

Objective 3 Examining time trading behaviour

Most of the respondents (1597; 88.8%) encountered at least one DCE_TTO_ task where duration differed between scenarios, and the time trading behaviour of respondents by the number of tasks they encountered is displayed in Table 4. Overall, 266 (16.7%) did not trade time in any of the tasks that they completed (i.e. always selected the longer duration irrespective of the number of tasks completed where duration differed), and 160 (10.0%) traded every time (i.e. selected the shorter duration for every task completed where duration varied). Therefore 1171 (75.8% of those completing at least two tasks with scenarios of different durations) displayed mixed trading behaviour (sometimes selecting the scenario with the longer duration and sometimes selecting the scenario with the shorter duration).

Note that if the scenario with the longer duration has more QALYs, then respondents are not expected to trade. Therefore, the 18 tasks with different durations for each scenario were ranked in terms of the gap in QALYs between the scenario with the longer duration and the scenario with the shorter duration: a negative gap indicates that the scenario with shorter duration has more QALYs. Table 5 presents this scenario ranking alongside the proportion of respondents selecting the scenario with the longer duration. The overall decreasing pattern observed is as expected: when the absolute difference in QALYs is large, a clear majority chooses the scenario with more QALYs; when the absolute difference is smaller, the margin becomes smaller. In three tasks, the majority fails to choose the scenario with more QALYs (rows 10, 12 and 13, in bold). Roughly speaking, where the absolute difference in QALYs is comparable, it does not seem the case that the split of responses is affected by whether the scenario with more QALYs has a shorter duration. So for example, tasks in rows 1, 17 and 18 have an absolute QALY gap of 4.1–4.5 QALYs, and these tasks have a roughly 80%–20% split of respondents in favour of the higher-QALY scenario, regardless of whether the scenario has longer or shorter duration. Similarly, rows 7 and 16 have an absolute QALY gap of 1.8, resulting in a 75%–25% split of respondents; or rows 8 and 15 have a QALY gap of 1.3 and a respondent split of 63%–37%. However, not all tasks follow this pattern.

Objective 4 The extent of agreement between individual ordinal preference and aggregate cardinal values

**Fig. 4:**
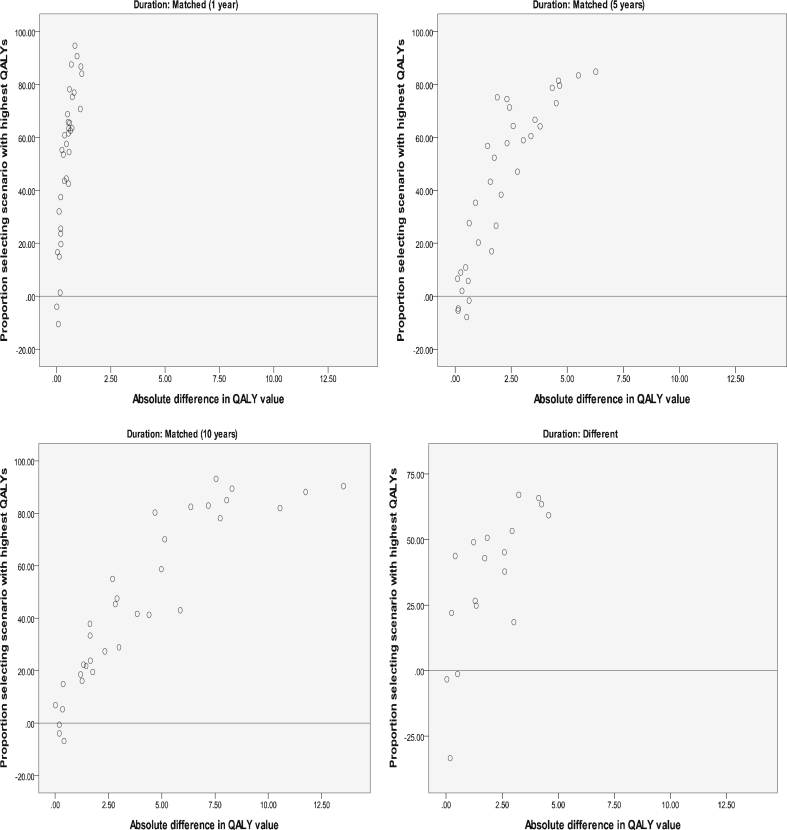
Percentage of respondents choosing higher QALY scenario by the absolute difference in QALYs (by scenario duration profile).

For each of the 120 DCE_TTO_ tasks, we examined the difference in the percentage of respondents choosing the profile with more QALYs over less, so that a positive figure indicates that the majority of respondents chose the scenario with more QALYs. If all respondents facing the same task choose the same scenario, this difference would be 100 − 0 = 100; if there is a 50%–50% split, then this difference would be 50 − 50 = 0. Fig. 4 plots this difference along the vertical axis against the absolute difference in implied QALYs along the horizontal axis, and this is done for four subsamples: sample 1–3 (top and left panels) are the tasks where duration is matched between scenarios, and sample 4 (bottom right panel) is the tasks where duration differs across scenarios. Across the four samples, most plots are in the positive range, and there is a rough positive correlation so that the further apart in terms of QALYs the two scenarios are, the larger is the proportion of those who choose the scenario with the higher QALYs.

For the matched one year sample, there is a group of tasks with very little difference in terms of QALYs, but a large difference in the response split across the tasks. This is because the difference in the health state values across the scenarios is large (between 0.67 and 1.16), but this large difference is not reflected in the difference in QALYs across the scenarios because they are only one year long (so the difference is due to the health state value rather than the overall amount of QALYs, and respondents consistently choose the less severe health state with the larger associated utility value.)

Across the samples, the tasks in the negative range of Fig. 4 indicate a disagreement between the ordinal preference and the implied cardinal values for the tasks. Of the 120 tasks, such a disagreement was observed in 12, all of them with very small difference in QALYs across the scenarios. For 11 of these, the difference between those choosing each scenario is 10% or less, indicating a low level of disagreement. For the one remaining task, however, the difference is large (33%) indicating a higher level of disagreement. This task consisted of scenario A with state 24144 for 5 years (−0.40QALYs) vs. scenario B with state 54514 for 1 year (−0.22QALYs). Of the five attributes: A and B are the same in two (Self care and Anxiety/depression); scenario A is better in two (Mobility and Usual activities); and scenario B is better in one (Pain/discomfort). In terms of QALYs, scenario B is better, but only a third of respondents agreed. The health state values of the respective health states are −0.08 for A and −0.22 for B, suggesting that there may be a fair proportion of respondents who perceive A is not worse than dead, whereas a larger proportion would agree that B is worse than dead. If a respondent believes scenario A is worse than dead, then five years of A may be less preferable than one year of B, so they may choose B. However, if a respondent believes A is better than dead then five years of A is more preferable to one year of B, so they choose A. Thus, a small variation in individual perception around dead (viz. slightly better than dead versus slightly worse than dead) for one scenario may lead to the opposite choice between the tasks.

Objective 5 Learning and fatigue effects across DCE_TTO_ questions

**Fig. 5:**
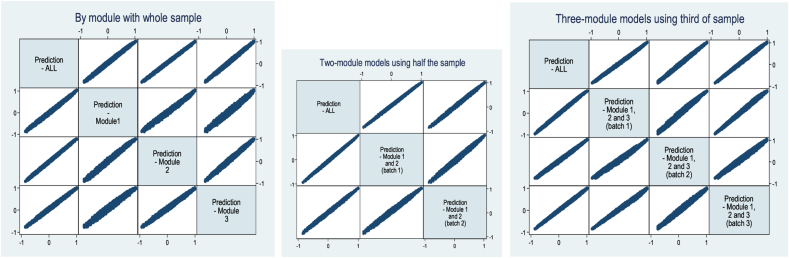
Scatter plots of the 3125 predicted values, by batch.

The correlation coefficients of the predicted EQ-5D-5L values across the batches are very high. For example, all the single-module batches have a correlation coefficient ranging from 0.985 for Module1(all) with Module3 (all) to 0.998 for ALL with Module2(all). A scatter plot matrix is given in the left hand panel of Fig. 5. The plots illustrate a very good direct correlation, with no bias by severity. This suggests that the three modules are each capturing similar preferences.

Table 3 presents the results for the restricted model which allows for scale difference across the three single-module batches. The size of estimated scale parameters increase negatively indicating that the error variance is increasing towards the end of the experiment. This suggests a fatigue effect. The LR statistic is 56.78, narrowly rejecting the null of preference homogeneity across the batches at the 5% significance level. The unrestricted models for each of the three single-module batches are also presented.

Objective 6 Comparing more DCE_TTO_ answers from a smaller sample and fewer DCE_TTO_ answers from a larger sample

From a visual inspection of the scatter plot matrices in Fig. 5, it can be seen that while there is little to choose between them, the double-module batches in the middle panel achieve the highest concentration of the plots, followed by the triple-module batches in the right hand panel. The relatively less concentrated scatter for the single-module batch Module 1 (All) suggests that asking a large sample of respondents five DCE_TTO_ questions may not be the most efficient way of administering the tasks.

Objective 7 The effect of the size of the sample

Fig. 5 suggests that the designs incorporating batches of 10 tasks with an intermediate sized sample, and 15 tasks with a smaller sample provide relatively stable results in comparison to the whole sample model.

## 4 Discussion

This study addressed two main research aims. For our first aim, results suggest that the DCE_TTO_ is a feasible method for generating health state utility values for larger descriptive systems such as that found in the EQ-5D-5L. DCE_TTO_ produces generally logically consistent coefficients across the levels within each the health state attribute, with only one coefficient (Mobility level 2) found to be non-significant (and disordered). The distribution of the predicted values for the 3125 health states is uni-modal, and there is no significant gap between the value for the best state (i.e. 11111), and the next best state (11211). This is in contrast to the EQ-5D MVH value set based on TTO where the distribution of the 243 predicted values is bi-modal, and there is a difference of 0.117 between the best (11111) and next best health state (11211). The five-level coefficients generated in this study are largely comparable with the three-level coefficients generated in Bansback et al. (2012). The only major difference in comparable levels between the three- and the five-level versions of EQ-5D was in the worst level of mobility. Since the wording has changed substantially, from “confined to bed” (EQ-5D mobility level 3) to “unable to walk about” (EQ-5D-5L mobility level 5), the fact that we found a difference gives some face validity to the method. Confidence intervals overlapped for all other comparable levels. We also found that a majority of respondents were willing to choose a health state with a shorter time frame, if the scenario with a shorter duration has a higher utility value. Finally, we found that for the majority of the tasks, there is agreement between the overall severity of the scenario pairs (expressed as the predicted value of the scenario in QALYs) and the health scenario chosen. This is important, as an individual patient may think health scenario, or prospect, A is better than B, but the population value set disagrees. When disagreement occurs it is generally because the overall QALY profile of both states is similar, or differences in respondents' perception of the states as better or worse than dead impacts on the frequencies choosing to live in the state given the associated duration. Robert and Dolan (2004) used the MVH dataset to assess agreement and found that for two thirds of respondents to agree with the ordinal ranking between two EQ-5D-3L health states, the cardinal difference between the states had to be as large as 0.20. Thus, a small variation in individual perception around dead (viz. slightly better than dead versus slightly worse than dead) for one scenario may lead to the opposite choice between the tasks.

For our second aim, although we found evidence of a fatigue effect, our analysis indicates that 10 or 15 DCE_TTO_ tasks may be better than five DCE_TTO_ tasks per respondent. In a recent comprehensive review, de Bekker-Grob et al. (2012) find that the mean number of choice sets per respondent in health-related DCEs is 14 and Bridges et al. (2011) suggest that including 8 to 16 choice tasks is good practice. However, this recommendation does not appear to have been based on empirical evidence which has mixed findings. For example Louviere et al. (2013) finds little impact on completion rates or response quality when asking 32 versus 16 choice tasks while Rose et al. (2009) found a large influence on response quality in one of the study groups when varying the number of choice sets from 9 to 15. Ultimately, fatigue effects will be associated with the complexity of the specific task – for example Louviere et al. (2013) finds that using more than two alternatives and more than 6 attributes had a larger influence on completion rates. Based on our findings, we are able to recommend that asking 10 to 15 DCE_TTO_ tasks is more efficient that asking more respondents fewer tasks, but do not rule out asking more than 15 tasks if time is not restricted. There has been limited formal work to establish the sample size requirements for DCEs (Orme, 2010). Most recently, Rose and Bliemer (2013) have proposed theoretically minimal sample size requirements. We should note however that an objective of many stated preference studies is to obtain values from a representative sample of a given population, and so there may be reasons beyond efficiency to include larger samples.

There are a number of limitations with the study design used which may impact on the findings presented. Firstly, only 18 of the 120 tasks had differing duration levels between the health scenarios. To predict utility values, the attribute coefficients are divided by the duration coefficient so any bias in the duration coefficient will bias the whole model. As can be seen, the confidence interval for the duration coefficient is large in comparison to the others, and it is possible that by increasing the number of tasks where duration varies, the size of the confidence interval could be reduced. At the same time, it should be borne in mind that the number of tasks in which duration may vary is limited by the fact that duration is interacted with the other attributes in the model. To identify the coefficients for these interactions, duration needs to be held constant within some tasks. Future developments of DCE_TTO_ should investigate the impact of increasing the number of tasks where duration differs. This may be achieved by basing the D-efficiency criterion on the covariance matrix of the anchored coefficients instead of the unanchored coefficients, as was done in the present study. Furthermore, it would also be possible to use a design that restricts implausible EQ-5D-5L states rather than manually checking the plausibility of the attribute combinations produced.

Secondly, there may be a number of concerns about the on-line methodology used (Mulhern et al., 2013). For example, the representativeness of the sample in terms of unobservable characteristics may be an issue. We also did not recruit respondents aged over 65 to take part, meaning that the sample is not fully representative of the adult population. However, our aim is not to develop a national tariff, but empirically test design issues relating to DCE_TTO_. It should also be noted that on-line panels give a highly cost effective way of achieving a large sample that is representative in terms of observable characteristics in a short space of time. Furthermore, there may be concerns about the fact that most commercial on-line panels offer financial rewards for completing surveys, and some panels allow members to participate in a large number of surveys. This may lead to poor quality responses, and it is up to the researcher to examine the relevant features of different on-line panels. Finally, there is limited information available about the level of respondent engagement with the task. The time taken to complete the survey was recorded, and a minimum completion time was set to attempt to include respondents who engaged with the tasks. However, no further information (or assurance) regarding engagement was available. This is likely to make on-line surveys that use iterative methods (such as TTO where an indifference value is identified for each state from each respondent) more vulnerable than those that use binary choice methods (such as DCE where each respondent only gives ordinal preferences).

Third, our analysis treated duration as a continuous variable assuming constant proportional time trade-off. Previous studies have found this assumption to hold at the aggregate level, but violated at the individual level (Tsuchiya and Dolan, 2005). We acknowledge this design assumption may have led to misspecification of the indirect utility function, contributing to the error in the model (de Bekker-Grob, 2013). Further exploration of model specification is possible by treating duration as a categorical variable (which the experimental design allows). This further analysis will allow the examination of whether preferences are linear in duration, and indeed whether the QALY model holds.

In conclusion, we find that the DCE_TTO_ is a feasible method that produces generally logically consistent coefficients for larger descriptive systems such as EQ-5D-5L. Analysis of data in batches indicates that completing 10 to 15 DCE_TTO_ tasks per respondent is better than completing five tasks. The impact of the total number of observations seems to be a more important factor affecting the stability of the coefficients than how a given number of total observations is allocated across different numbers of respondents.

## Figures and Tables

**Table 1:**
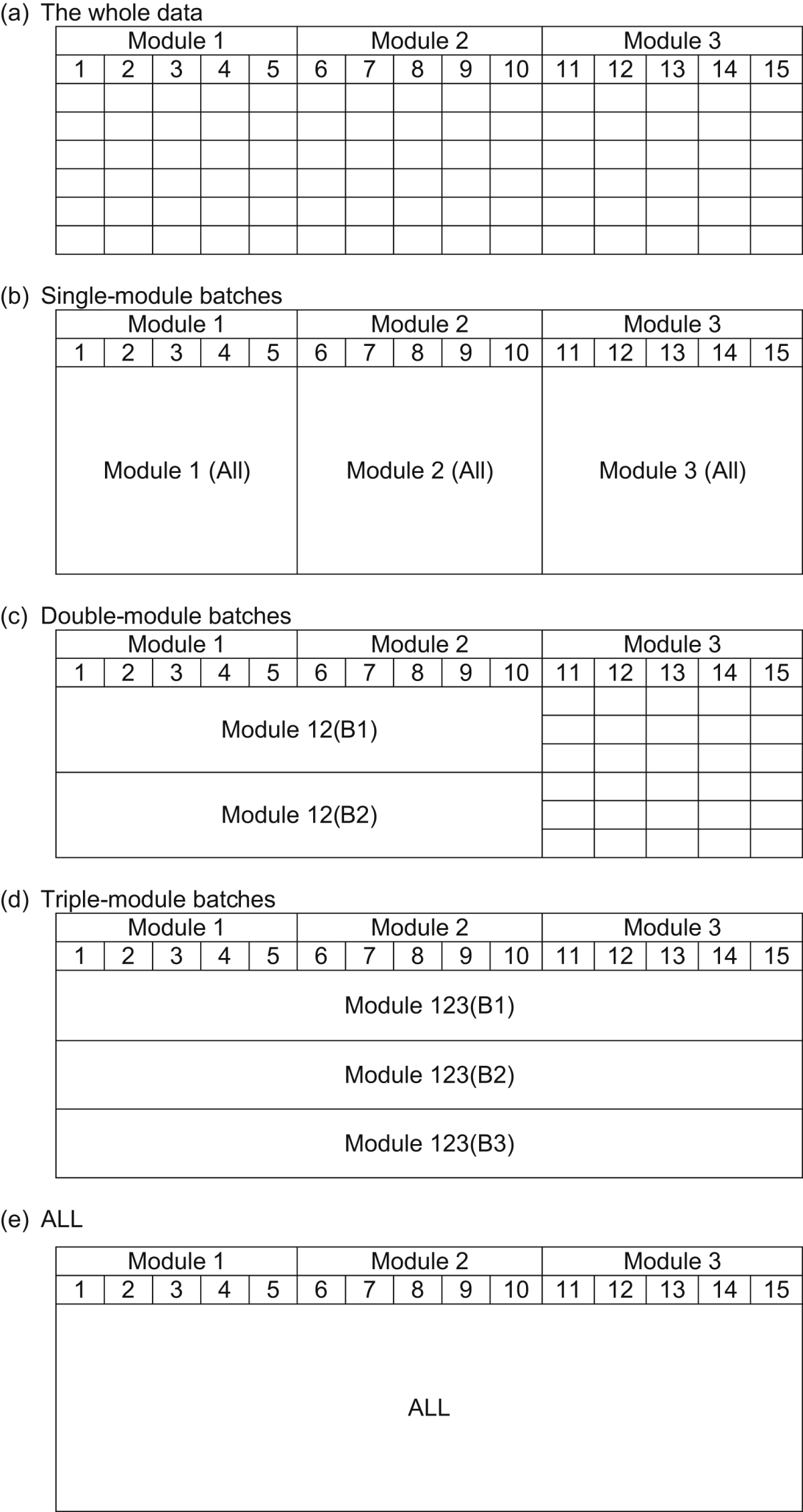
A stylised representation of the study design.

**Table 2:** Background characteristics.

Characteristic	Sample	General population^e^
*n*	1799	
Female	54%	52%
**Age**		
18–30	30%	26%
31–50	44%	45%
51–66	26%	29%
Mean (SD)	40 (13)	42%
**Employment status**		
In employment^a^	58%	62%
Student	9%	7%
Not working^b^	17%	21%
**Marital status**		
Married or with partner	57%	53%
**Education level**		
Minimum school leaving age	22%	n/a
Degree or equivalent	42%	22%
**Self reported health and satisfaction**		
In good health^c^	77%	n/a
Satisfied with health^d^	67%	n/a
Satisfied with life^d^	66%	n/a

a Employed or self employed.

b Seeking work; Unemployed; or Long-term sick.

c Excellent, Very good, or Good self reported own health.

d Score 6 or above on a scale of 0–10.

e General population stats extracted from the UK Census 2001 (ONS, 2005).

**Table 3:** DCE_TTO_ coefficients overall.

	Whole sample	Unrestricted model			Restricted model
	ALL	Module 1	Module 2	Module 3	ALL
No.DCE	15	5	5	5	15
M2xY	0.006	0.014	0.001	0.002	0.006
M3xY	−0.021***	−0.008	−0.028***	−0.027***	−0.022***
M4xY	−0.096***	−0.086***	−0.095***	−0.107***	−0.102***
M5xY	−0.116***	−0.1257***	−0.117***	−0.110***	−0.125***
SC2xY	−0.004	0.002	0.002	−0.016*	−0.004
SC3xY	−0.022***	−0.024**	−0.016**	−0.026***	−0.024***
SC4xY	−0.103***	−0.114***	−0.104***	−0.095***	−0.111***
SC5xY	−0.133***	−0.152***	−0.123***	−0.129***	−0.144***
UA2xY	−0.025***	−0.037***	−0.019**	−0.021**	−0.027***
UA3xY	−0.048***	−0.057***	−0.049***	−0.040***	−0.052***
UA4xY	−0.082***	−0.088***	−0.087***	−0.074***	−0.088***
UA5xY	−0.092***	−0.107***	−0.095***	−0.077***	−0.099***
PD2xY	−0.027***	−0.040***	−0.027***	−0.016*	−0.029***
PD3xY	−0.064***	−0.081***	−0.065***	−0.049***	−0.070***
PD4xY	−0.155***	−0.167***	−0.153***	−0.149***	−0.167***
PD5xY	−0.197***	−0.216***	−0.191***	−0.188***	−0.212***
AD2xY	−0.033***	−0.047***	−0.025**	−0.029***	−0.036***
AD3xY	−0.065***	−0.081***	−0.058***	−0.059***	−0.071***
AD4xY	−0.169***	−0.208***	−0.156***	−0.149***	−0.184***
AD5xY	−0.189***	−0.207***	−0.190***	−0.174***	−0.204***
Y	0.393***	0.431***	0.372***	0.384***	0.424***
Log of scale parameter – module 2					−0.083***
Log of scale parameter – module 3					−0.144***
LL statistic	−15,813.054	−5162.8781	−5281.012	−5334.5721	−15806.85
Observations	26,985	8995	8995	8995	26,985

*p < 0.1; **p < 0.05; ***p < 0.001.

**Table 4:** Trading behaviour on duration at the overall level.

*N* of tasks where duration differs	*N* survey versions	*N* completing	*N* (%) never trading	*N* (%) always trading	*N* (%) mixed trading
1	1	52	30 (57.7)	22 (42.3)	n/a
2	1	50	8 (16.0)	11 (22.0)	31 (62.0)
3	19	944	159 (16.8)	104 (11.0)	681 (72.1)
4	6	299	32 (10.7)	11 (3.7)	256 (85.6)
5	2	101	13 (12.9)	7 (6.9)	81 (80.2)
6	3	151	24 (15.9)	5 (3.3)	122 (80.8)
ALL	31	1597	266 (16.7)	160 (10.0)	1171 (75.8^1^)

Percentage of respondents who are able to display mixed trading behaviour (i.e. completing at least 2 pairs where duration differs (*n* = 1545)).

**Table 5:** Trading behaviour across the 18 tasks where duration differs (ordered by QALY difference (longer duration − shorter duration).

Row ID	QALY difference	% Choosing larger QALY	% Choosing shorter duration	Scenario with longer duration					Scenario with shorter duration			
				Obs	State	*T*	Tariff	QALY	State	*T*	Tariff	QALY
1	4.25	81.73	18.27	301	15212	10	0.516	5.159	42224	5	0.181	0.905
2	3.24	83.50	16.50	309	53332	10	0.277	2.773	42555	1	−0.467	−0.467
3	3.02	59.25	40.75	292	33251	10	0.323	3.232	25241	1	0.208	0.208
4	2.94	76.61	23.39	295	41523	10	0.288	2.884	51335	1	−0.060	−0.060
5	2.61	68.87	31.13	302	51141	10	0.307	3.070	33114	1	0.456	0.456
6	2.61	72.55	27.45	306	42124	10	0.245	2.454	23155	5	−0.030	−0.151
7	1.72	71.43	28.57	294	13314	5	0.393	1.967	42151	1	0.243	0.243
8	1.29	63.30	36.70	297	42531	5	0.348	1.739	21143	1	0.445	0.445
9	1.22	74.49	25.51	294	25141	5	0.272	1.360	45421	1	0.140	0.140
**10**	**0.51**	**49.35**	**50.65**	**306**	**24551**	**10**	**0.009**	**0.094**	**23444**	**5**	**−0.083**	**−0.415**
11	0.41	71.85	28.15	302	41515	5	0.041	0.207	54414	1	−0.200	−0.200
**12**	**0.03**	**48.34**	**51.66**	**302**	**42324**	**10**	**0.123**	**1.231**	**35,332**	**5**	**0.240**	**1.199**
**13**	**−0.18**	**33.33**	**33.33**	**294**	**24144**	**5**	**−0.080**	**−0.402**	**54514**	**1**	**−0.224**	**−0.224**
14	−0.25	61.00	61.00	300	55223	5	0.066	0.332	13332	1	0.580	0.580
15	−1.34	62.37	62.37	295	24153	10	0.078	0.776	14512	5	0.423	2.114
16	−1.83	75.33	75.33	304	31455	5	−0.240	−1.202	32232	1	0.623	0.623
17	−4.13	82.89	82.89	298	34435	10	−0.165	−1.652	31333	5	0.495	2.477
18	−4.57	79.61	79.61	304	15445	10	−0.413	−4.128	14223	1	0.443	0.443

Tasks where scenario with lower QALYs is chosen by the majority are in bold.
